# Change in sensitivity to visual error in superior colliculus during saccade adaptation

**DOI:** 10.1038/s41598-017-10242-z

**Published:** 2017-08-29

**Authors:** Yoshiko Kojima, Robijanto Soetedjo

**Affiliations:** Department of Physiology and Biophysics, Washington National Primate Research Center, University of Washington, Seattle, Washington, Washington, 98195-7330 USA

## Abstract

Saccadic eye movements provide a valuable model to study the brain mechanisms underlying motor learning. If a target is displaced surreptitiously while a saccade is underway, the saccade appears to be in error. If the error persists gradual neuronal adjustments cause the eye movement again to land near the target. This saccade adaptation typically follows an exponential time course, i.e., adaptation speed slows as adaptation progresses, indicating that the sensitivity to error decreases during adaptation. Previous studies suggested that the superior colliculus (SC) sends error signals to drive saccade adaptation. The objective of this study is to test whether the SC error signal is related to the decrease in the error sensitivity during adaptation. We show here that the visual activity of SC neurons, which is induced by a constant visual error that drives adaptation, decreases during saccade adaptation. This decrease of sensitivity to visual error was not correlated with the changes of primary saccade amplitude. Therefore, a possible interpretation of this result is that the reduction of visual sensitivity of SC neurons contributes an error sensitivity signal that could help control the saccade adaptation process.

## Introduction

For goal directed tasks, the brain keeps the movement accurate by reducing its error. This process is called motor adaptation or motor learning. The error, i.e., the distance between the goal and the end point of the actual movement, is usually detected visually and guides the correction of the motor command.

Saccades provide an excellent model to study the neuronal mechanisms of motor adaptation because the basic circuitry for saccade generation is well studied^1^. Moreover, it is possible to induce an apparent error by displacing the target during a saccade so the saccade appears to have fallen short or to have overshot^2^. If the error persists, the oculomotor system gradually adjusts the signal that is producing the faulty saccade so the saccade lands closer to the displaced target. This process is called saccade adaptation. Saccade adaptation typically follows an exponential time course. That is, adaptation speed slows as adaptation progresses^3, 4^ and this might be due to a decrease in the sensitivity to error^5, 6^ during adaptation. The signal that might be instrumental in controlling this sensitivity to error has not been elucidated.

A recent body of research suggests that the cerebellum plays an important role in saccade adaptation. Cerebellar learning theory^7, 8^ suggests that when a movement is inaccurate, the resultant error increases the complex spike activity of Purkinje cells (P-cells). The increased complex spike activity, in turn weakens the synaptic strength of the parallel fibers on P-cells to decrease their simple spike activity. This altered simple spike activity then influences motor commands in the brainstem or elsewhere. Consistent with this theory, the probability of complex spike occurrence in the oculomotor vermis (OMV) increases and the frequency of simple spikes decreases during saccade adaptation (refs 9–12, cf. refs 13 and 14).

Two lines of research have implicated the superior colliculus (SC) as one possible source of the complex spikes associated with the error signal to drive saccade adaptation. First, there are well-demonstrated disynaptic routes from the SC to the OMV. The climbing fibers that cause complex spikes in the OMV P-cell originate in the inferior olive^15, 16^, which receives a projection from the SC^17, 18^. Second, stimulation of the rostral SC timed to the occurrence of complex spike enhancement during saccade adaptation actually drives saccade adaptation without any “natural” visual error^19, 20^. This finding suggests that rostral SC stimulation can act as a surrogate error signal to drive adaptation, presumably by evoking complex spikes in the OMV.

But what kind of information does the SC signal provide during saccade adaptation? In particular, does the SC visual signal encode only the size of the visual error or the sensitivity to error? To address this question, we asked whether the sensitivity to visual error of SC neurons changes during adaptation. With traditional paradigms^2^, adaptation is induced by a forward or backward target jump during a targeting saccade. Because the size of the target jump is constant, the saccade amplitude changes produced by adaptation reduce this imposed visual error. To reveal whether SC neurons have a signal related to the change in visual error sensitivity during adaptation, we used an adaptation paradigm that held the error size constant^3, 4^. Any changes in the SC visual response during constant visual error adaptation must then be attributed to a change in visual sensitivity. If SC visual activity encodes only the size of the visual error, we expect that the visual activity will not change during this adaptation paradigm. However, if it encodes the *sensitivity* to error, we expect a decrease in the visual activity during adaptation.

We find that the firing rate of the visual component of many SC neurons decreased during adaptation. Furthermore, the visual activity decreased during both amplitude decrease and increase adaptations, indicating that the change in the primary saccade metrics is not the reason for the decrease in the visual activity. Moreover, the SC burst associated with corrective saccades did not change during adaptation, indicating that the decrease in visual activity was not due to a decrease in general excitability over time. These data are consistent with the possibility that the SC might be involved in reducing the error sensitivity during saccade adaptation.

## Results

### Amplitude decrease adaptation - visuo-motor neurons

**Figure 1:**
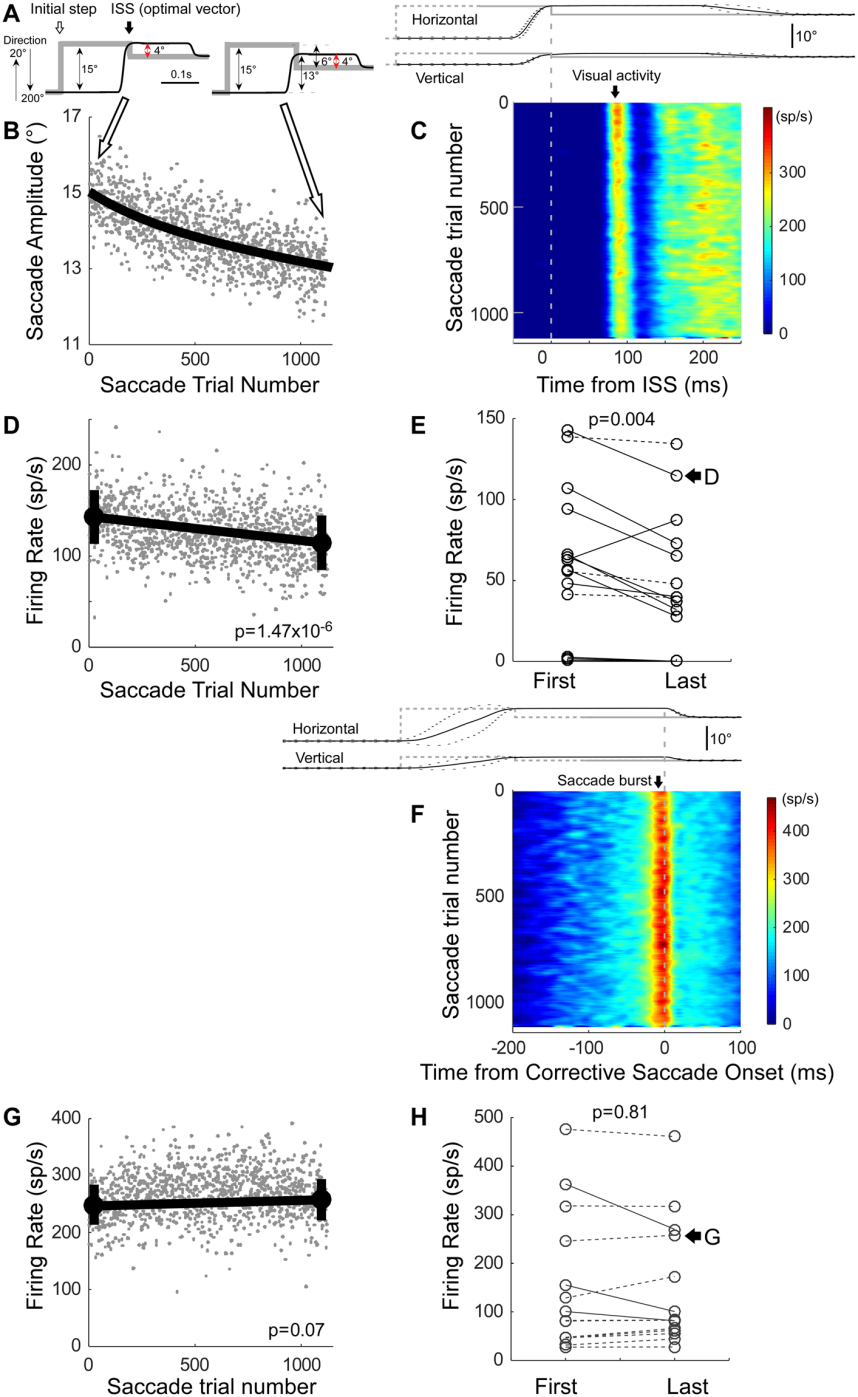
Activity of visuo-motor neurons during amplitude decrease adaptation. (**A**–**D**) Visual activity of a representative neuron in the right SC of monkey-D. (**A**) Schematic of target (gray lines) and eye (black lines) movements for the beginning and end of adaptation. Target stepped along the neuron’s optimal direction, in this case 200° (rightward: 0°, upward: 90°, leftward: 180°, downward: 90°). During adaptation, the visual error was held constant at 4° (red arrow), i.e., this neuron’s optimal amplitude. (**B**) Decrease in primary saccade amplitude with saccade trial number. Grey dots represent individual saccades; black line is an exponential fit. (**C**) Heat map of visual activity during adaptation aligned on ISS onset (time = 0). Upper panel, target (grey) and eye movements (black) for the first 15 saccades during adaptation aligned on ISS onset at end of the primary saccade. Black broken lines indicate 1 standard deviation (SD) of eye movement; grey broken lines approximate the time of the initial target step (because the latency of the primary saccade is variable). (**D**) Average firing rate of the visual response for individual trials during adaptation (grey dots) and the means of the first and last 50 saccades (black circles, error bars are 1 SD). (**E**) Mean firing rates of visual activity for 14 visuo-motor neurons. Lines connect the data from each neuron. Solid line indicates a significant decrease, dotted line indicates no significant decrease. (**F**–**H**) Saccade-related burst activity. (**F,G**) The representative neuron in A–D. (**F**) Heat map of saccade-related burst aligned on corrective saccade onset. Upper panel, target (grey) and eye movements (black) for first 15 saccades during adaptation aligned on corrective saccade onset. (**G**) Average burst firing rate during adaptation. (**H**) Mean firing rate of saccade-related activity for the 14 visuo-motor neurons.

Figure 1A–D consider the visual activity of a representative visuo-motor neuron during amplitude decrease adaptation. For this neuron, the optimal visual receptive field direction was 200°, i.e., to the left and slightly downward and its optimal amplitude was 4°. We adapted saccades to target steps of 15° in the opposite direction of the optimal direction, i.e., 20°, with a 4° backward intra-saccadic step (ISS) visual error (Fig. 1A, see Methods for details). Saccade amplitude gradually decreased (Fig. 1B). The adaptation followed an exponential time course, i.e., adaptation rate was faster at beginning and slower at end. Figure 1C shows the visual activity of the SC neuron aligned on the occurrence of the ISS (time 0). Although, we kept the visual error at the end of the primary saccades fairly constant at 4° throughout the entire adaptation session (Fig. 1A, see Methods for details), the visual activity decreased (Fig. 1C, black arrow with “Visual activity”) (see also Supplementary Fig. S1). Figure 1D plots the average firing rate in the interval between 50 and 100 ms^21, 22^ after the ISS. The mean firing rate associated with the last 50 saccade trials (114.5 ± 30.0) was significantly less than that associated with the first 50 saccade trials (142.8 ± 29.6) (black circles and error bars, Wilcoxon rank sum test, p = 1.47 × 10^−6^).

For the population of 14 visuo-motor neurons (Fig. 1E), the mean visual activity associated with the last 50 saccade trials was significantly less than that associated with the first 50 saccade trials (Wilcoxon signed rank test, p = 0.0040). Eleven of fourteen individual neurons (79%) showed significantly less activity for the last 50 saccades than for the first 50 (Wilcoxon rank sum test, p < 0.05).

Figure 1F,G shows the saccade-related burst activity of the visuo-motor neuron in Fig. 1A–D aligned on the onset of the corrective saccade. The burst did not change during adaptation (Fig. 1F). Figure 1G plots the average burst rate from 8 ms before until 8 ms^21^ after each corrective saccade. There was no significant difference in the average burst rate associated with the first and last 50 saccades (Wilcoxon rank sum test, p = 0.07).

For the population of 14 visuo-motor neurons (Fig. 1H), the mean saccade-related burst rates associated with the first and last 50 saccades were not significantly different (Wilcoxon signed rank test, p = 0.81). Only 3/14 individual neurons (21%) showed significantly (Wilcoxon rank sum test, p < 0.05) less activity for the last 50 saccades than for the first 50.

### Amplitude decrease adaptation - visual neurons

**Figure 2:**
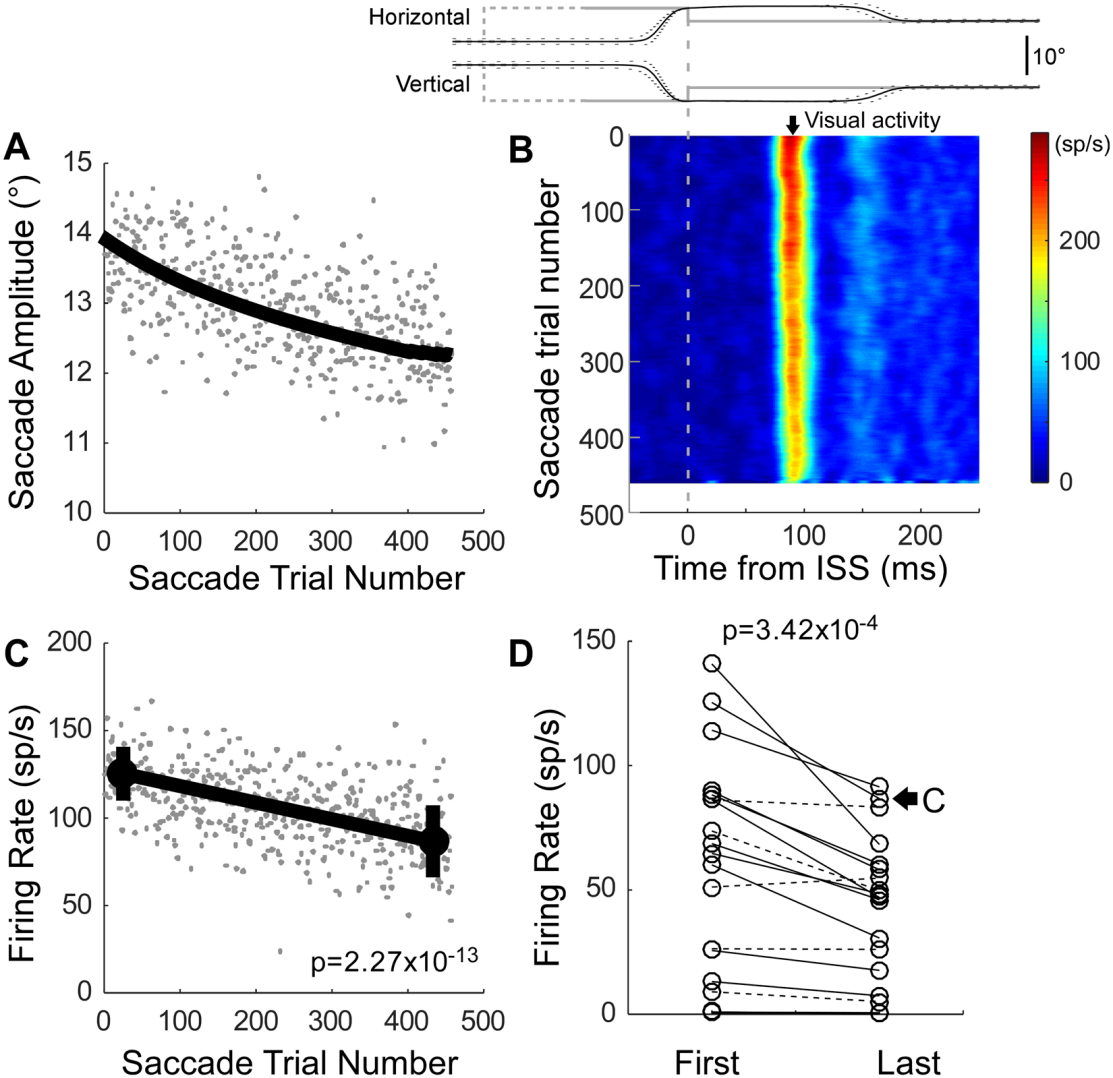
Activity of visual neurons during amplitude decrease adaptation. (**A**–**D)** Analyses like those of Fig. 1B–E. (**A**–**C**) Visual activity of a representative neuron in the right SC of monkey-D, the neuron’s optimal direction was 200°, amplitude was 3°.

A decrease in the visual response during adaptation was demonstrable for all unit types with visual responses. For the exemplar visual neuron in Fig. 2A–C, the visual activity (Fig. 2B) decreased gradually during amplitude decrease adaptation (Fig. 2A). The mean firing rate associated with the last 50 saccade trials was significantly less than that associated with the first 50 (Wilcoxon rank sum test, p = 2.27 × 10^−13^) (Fig. 2C). For the population of 19 visual neurons, the activity associated with the last 50 saccade trials was significantly less than that associated with the first 50 (Wilcoxon signed rank test, p = 3.42 × 10^−4^). Fourteen of nineteen individual neurons (74%) showed significantly (Wilcoxon rank sum test, p < 0.05) less activity associated with the last 50 saccades than with the first 50.

### Amplitude decrease adaptation – saccade-related burst neurons

**Figure 3:**
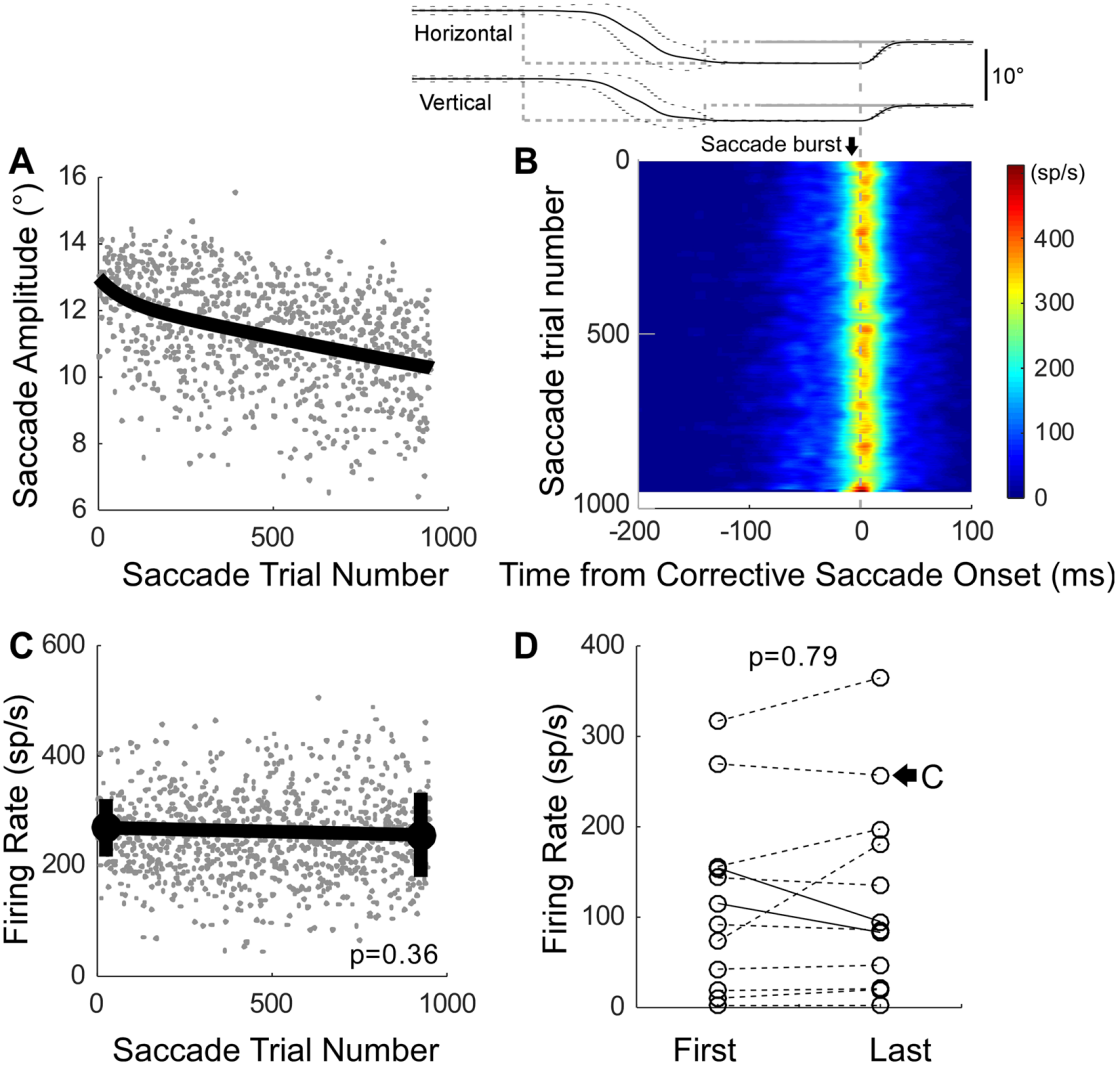
Activity of saccade-related burst neurons during amplitude decrease adaptation. (**A–D**) Analyses like those of Fig. 1B, F–H. (**A**–**C**) Saccade-related burst activity of a representative neuron of left SC in monkey L, the neuron’s optimal direction was 40°, amplitude was 8°.

Figure 3A–C shows activity from a representative saccade-related burst neuron. The saccade-related activity showed little qualitative change during adaptation (Fig. 3A,B). The mean burst rate of the last 50 saccades was not significantly different from that of the first 50 (Wilcoxon rank sum test, p = 0.36) (Fig. 3C). For the entire population of saccade-related burst neurons, the mean burst rates associated with the first and last 50 saccades (Fig. 3D) were not significantly different (Wilcoxon signed rank test, p = 0.791). Only 3/12 (25%) individual neurons showed significantly (Wilcoxon rank sum test, p < 0.05) less activity for the last 50 saccades than the first 50.

### Amplitude decrease adaptation – population average

**Figure 4:**
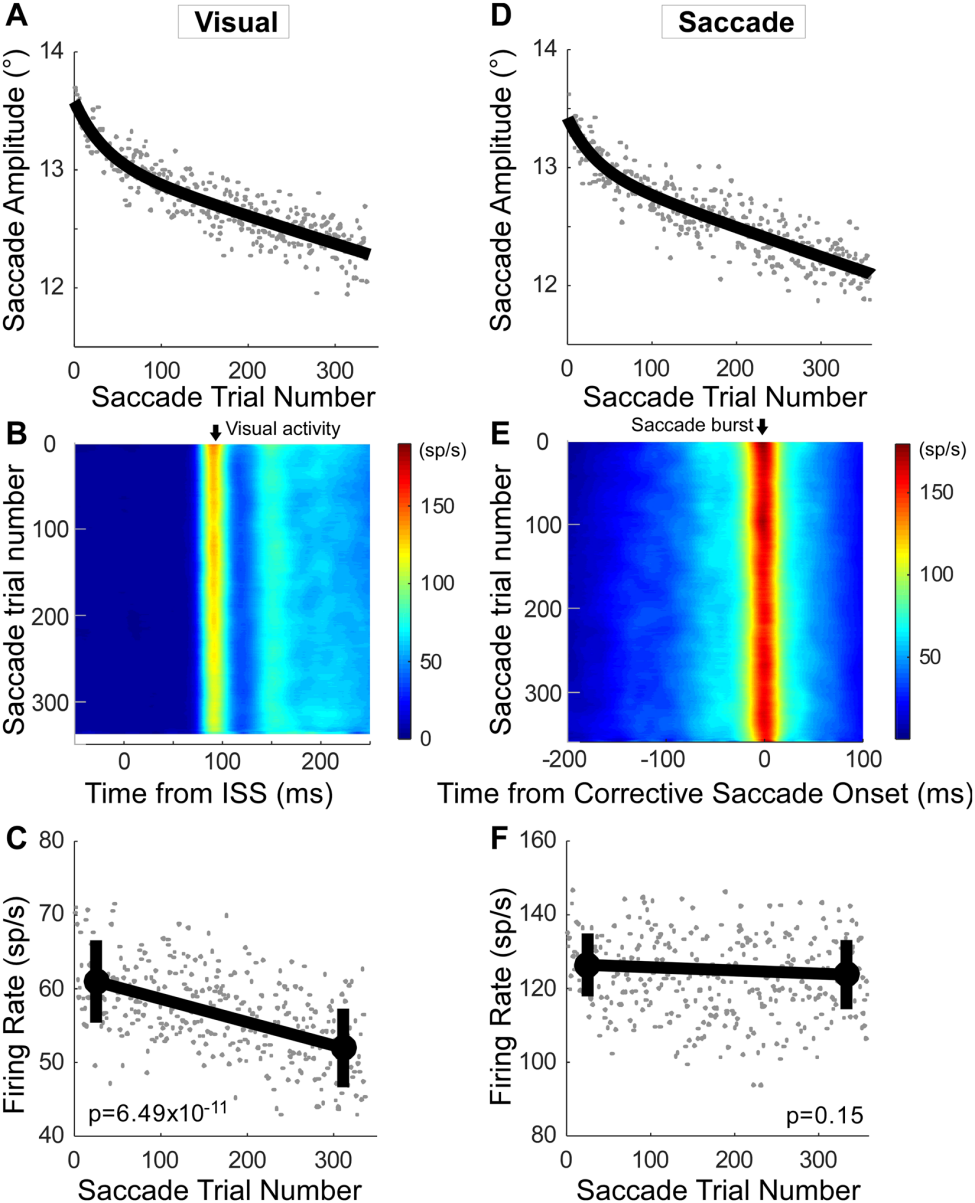
Summary of visual and saccade-related activity changes during amplitude decrease adaptation. (**A**–**C**) Population average of visual activity for 37 neurons. (**A**) Population change in saccade amplitude during adaptation with exponential fit. (**B**) associated change in average visual activity aligned on ISS occurrence (0 ms). (**C**) Average firing rate and the mean of the first and last 50 saccades (±1 SD). (**D**–**F**) Population average of saccade burst activity for 35 neurons. (**D**) Population change in saccade amplitude during adaptation with exponential fit. (**E**) Associated change in saccade-related burst activity aligned on corrective saccade onset. (**F**) Average firing rate and the mean of the first and last 50 saccades (±1 SD).

Figure 4 shows the population average of visual activity (Fig. 4A–C) and saccade-related burst activity (Fig. 4D–F) during amplitude decrease adaptation. For visual activity, we averaged the data across visual (n = 14), visuo-motor (n = 19) and buildup neurons (n = 4). For these 37 neurons, each data set consisted of at least 336 saccade trials; therefore we averaged data from only the first to the 336th saccade from each experiment. The population average of the saccade amplitude was well fit with an exponential function (r^2^ = 0.82), indicating that adaptation was faster at the beginning and slower at the end (Fig. 4A). The visual activity decreased gradually (Fig. 4B). The mean visual firing rate associated with the last 50 saccade trials was significantly less than that associated with the first 50 (Wilcoxon rank sum test, p = 6.49 × 10^−11^) (Fig. 4C).

In contrast, the saccade-related burst did not change during adaptation. For neurons that exhibited a saccade-related burst, i.e., visuo-motor (n = 19), saccade-related burst (n = 12) and buildup neurons (n = 4), each data set had at least 358 saccade trials. Over the 358 trials in each data set, the adaptation speed of the population average was faster at the beginning and slower at the end (Fig. 4D), but the saccade-related burst did not change (Fig. 4E). The mean burst rate of the first and last 50 saccades was not significantly different (Wilcoxon rank sum test, p = 0.15) (Fig. 4F).

### Amplitude increase adaptation – population average

For amplitude increase adaptation, the primary and corrective saccades are in the same direction (see Methods); therefore, the number of adapted trials was always less for gain increase than gain decrease adaptation. We discarded the datasets with less than 100 saccade trials.

**Figure 5:**
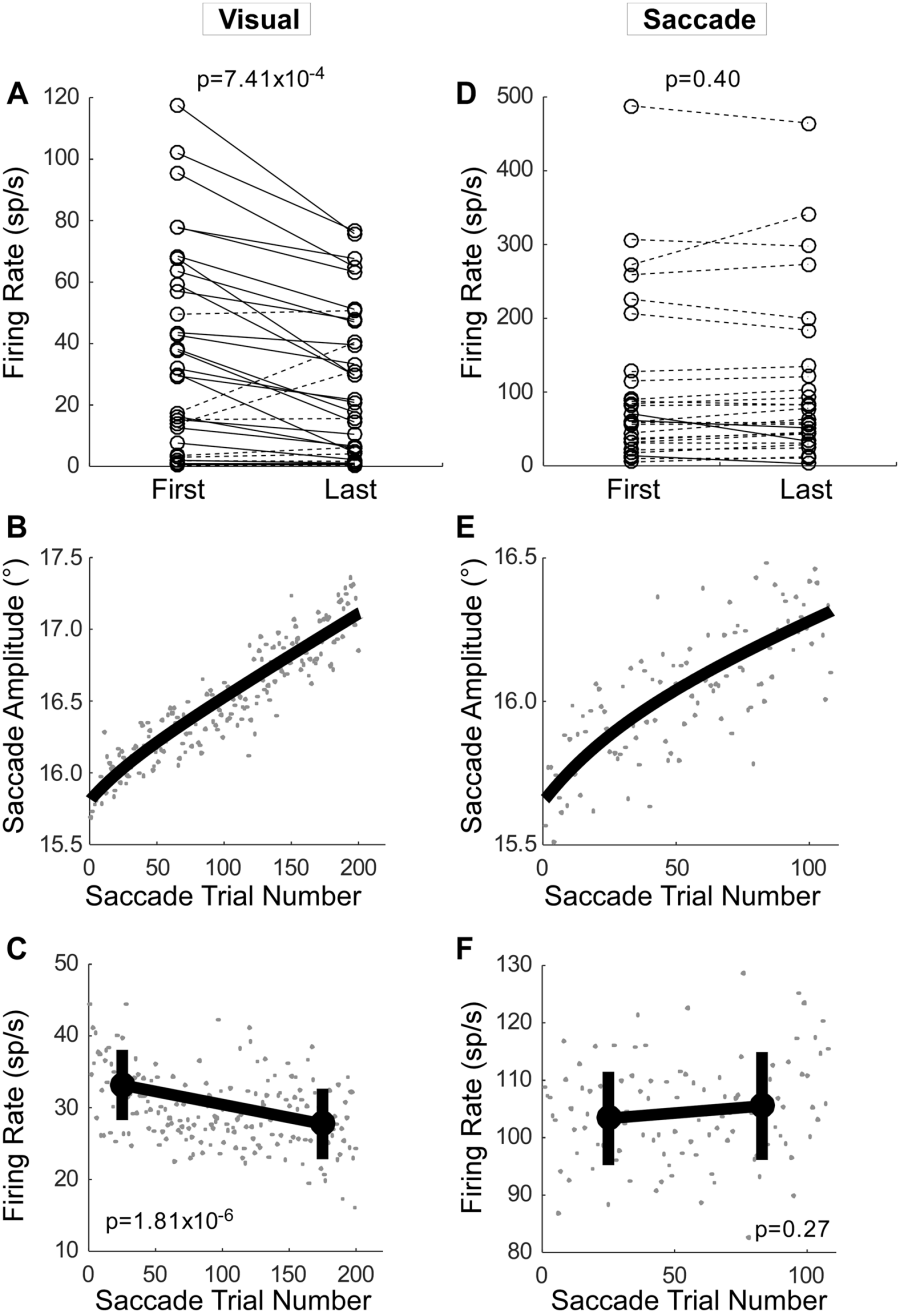
Summary of visual and saccade-related activity changes during amplitude increase adaptation. (**A**–**C**) Population average of visual activity for 35 neurons. (**D**–**F**) Population average of saccade related burst activity for 30 neurons. (**A**,**D**) Mean firing rates of first and last 50 saccades during adaptation for individual neurons. (**B**,**E**) Population change in saccade amplitude during adaptation with exponential fit. (**C**,**F**) Average firing rate and the mean of the first and last 50 saccades (±1 SD) for the visual and saccade burst activity, respectively.

A decrease in the visual response during adaptation also was demonstrable for amplitude increase adaptation. Figure 5A summarizes the mean visual firing rate associated with the first and last 50 saccade trials for all 35 neurons with visual activity. The visual activity associated with the last 50 saccades was significantly less than that associated with the first 50 for all neurons (Wilcoxon signed rank test, p = 7.41 × 10^−4^). Twenty three of thirty five individual neurons (66%) showed significantly (Wilcoxon rank sum test, p < 0.05) less activity associated with the last 50 saccades than with the first 50.

For these 35 neurons, each data set consisted of at least 200 saccade trials; therefore we averaged data from only the first to the 200th saccade number from each experiment to calculate the population average. The population averages showed an exponential increase in amplitude and a consequent slowing in adaptation speed (Fig. 5B), and a decrease in firing rate (Fig. 5C). The average firing rate associated with the last 50 saccade trials was significantly less than that associated with the first 50 (Wilcoxon rank sum test, p = 1.81 × 10^−6^) (Fig. 5C).

In contrast, for the 30 neurons with saccade-related activity, the average burst rate was not significantly different for the first and last 50 saccades (Fig. 5D; Wilcoxon signed rank test, p = 0.40). Only three of thirty (10%) showed significantly (Wilcoxon rank sum test, p < 0.05) less activity for the last 50 saccades than the first 50. Each data set contained at least 108 saccade trials and for this number of trials, the population average burst rate for the first and last 50 saccades was not significantly different for any neuron (Wilcoxon rank sum test, p = 0.27) (Fig. 5F).

### Measuring other parameters

**Figure 6:**
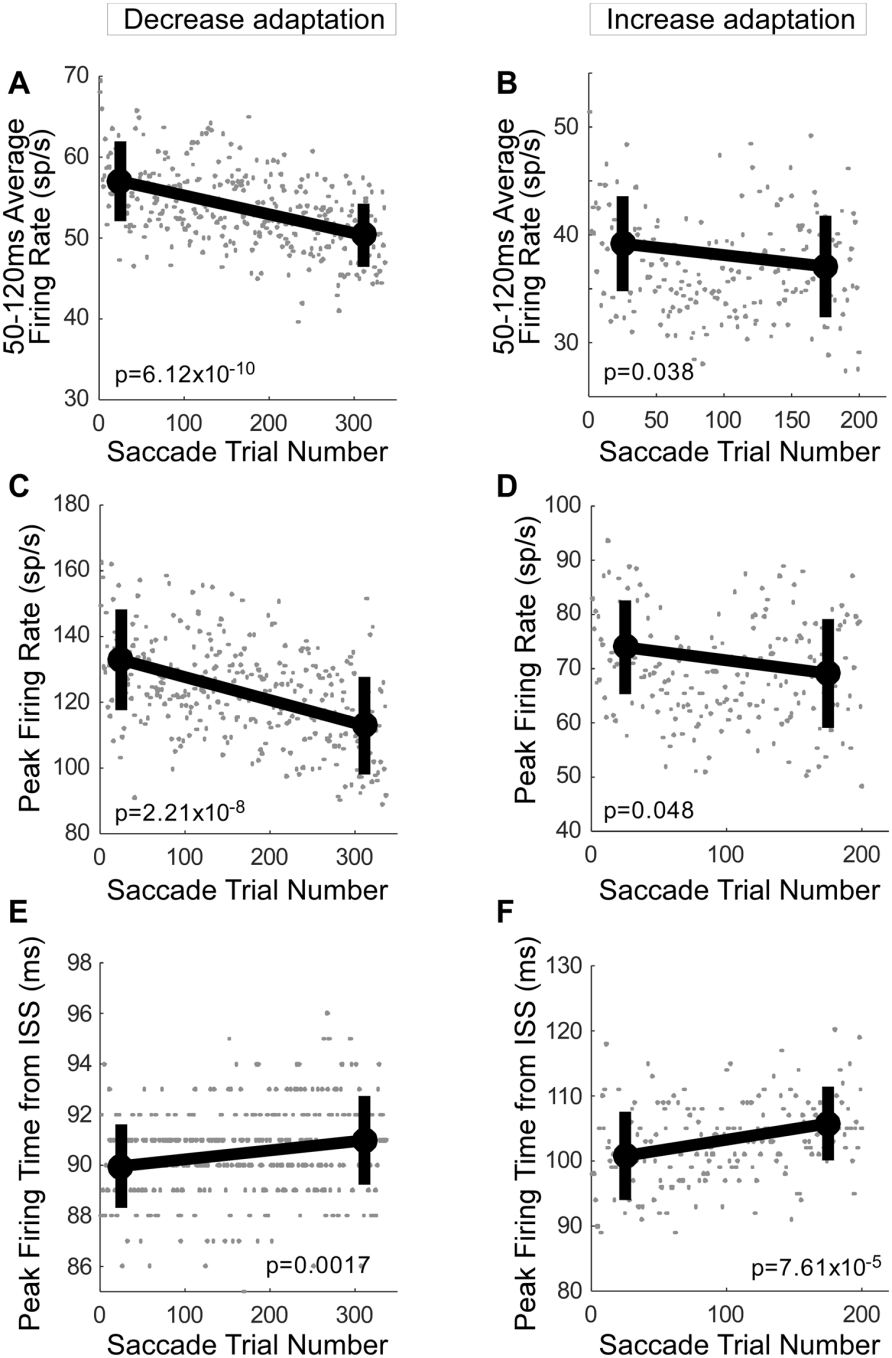
Population averages for three other measurements of visual activity. (**A**,**C**,**E**) Population average for the 37 neurons of Fig. 4A–C during amplitude decrease adaptation. (**B**,**D**,**F**) Population average for the 35 neurons of Fig. 5A–C during increase adaptation. (**A**,**B**) Average firing rate in the interval between 50 and 120 ms after ISS during adaptation. (**C**,**D**) Average peak firing rate in the interval between 50 and 120 ms after ISS. (**E**,**F**) The time to peak firing in the interval between 50 and 120 ms after ISS. Thick lines connect the mean measures of the first and last 50 saccade trials (±SD).

The decrease in the visual activity with adaptation did not depend on how the visual activity was measured. Thus far, we have considered the activity in the interval from 50 to 100 ms^21, 22^ after the onset of an ISS as the visual response. However, a 100 ms limit cuts off the tail of the visual activity in our experiments (Fig. 4B). Therefore, we also measured the average firing rate between 50 and 120 ms after the onset of an ISS^23^. For these re-binned data, the population average of the visual activity also decreased for both amplitude decrease (Fig. 6A) and increase adaptation (Fig. 6B). The average firing rate associated with the last 50 saccade trials was significantly less than that associated with the first 50 in both adaptation directions (Wilcoxon rank sum test, decrease adaptation: p = 6.12 × 10^−10^, Fig. 6A; increase: p = 0.04, Fig. 6B).

The same significant decrease occurred when the peak rate in the 50–120 ms interval was taken as the visual response measure (see Fig. 4B). The average maximum firing rate for the last 50 saccade trials was significantly less than for the first 50 in both adaptation direction (Wilcoxon rank sum test, decrease adaptation: p = 2.21 × 10^−8^, Fig. 6C, increase: p = 0.048, Fig. 6D).

The time of peak firing was significantly delayed for both amplitude decrease (Fig. 6E) and increase (Fig. 6F) adaptation. The average peak firing time for the last 50 saccade trials was significantly later than for the first 50 (Wilcoxon rank sum test, decrease adaptation: p = 0.0017, Fig. 6E, increase: p = 7.61 × 10^−5^, Fig. 6F). Therefore, the visual activity became both smaller and occurred later as adaptation progressed.

### Control analysis – primary saccade amplitude

**Figure 7:**
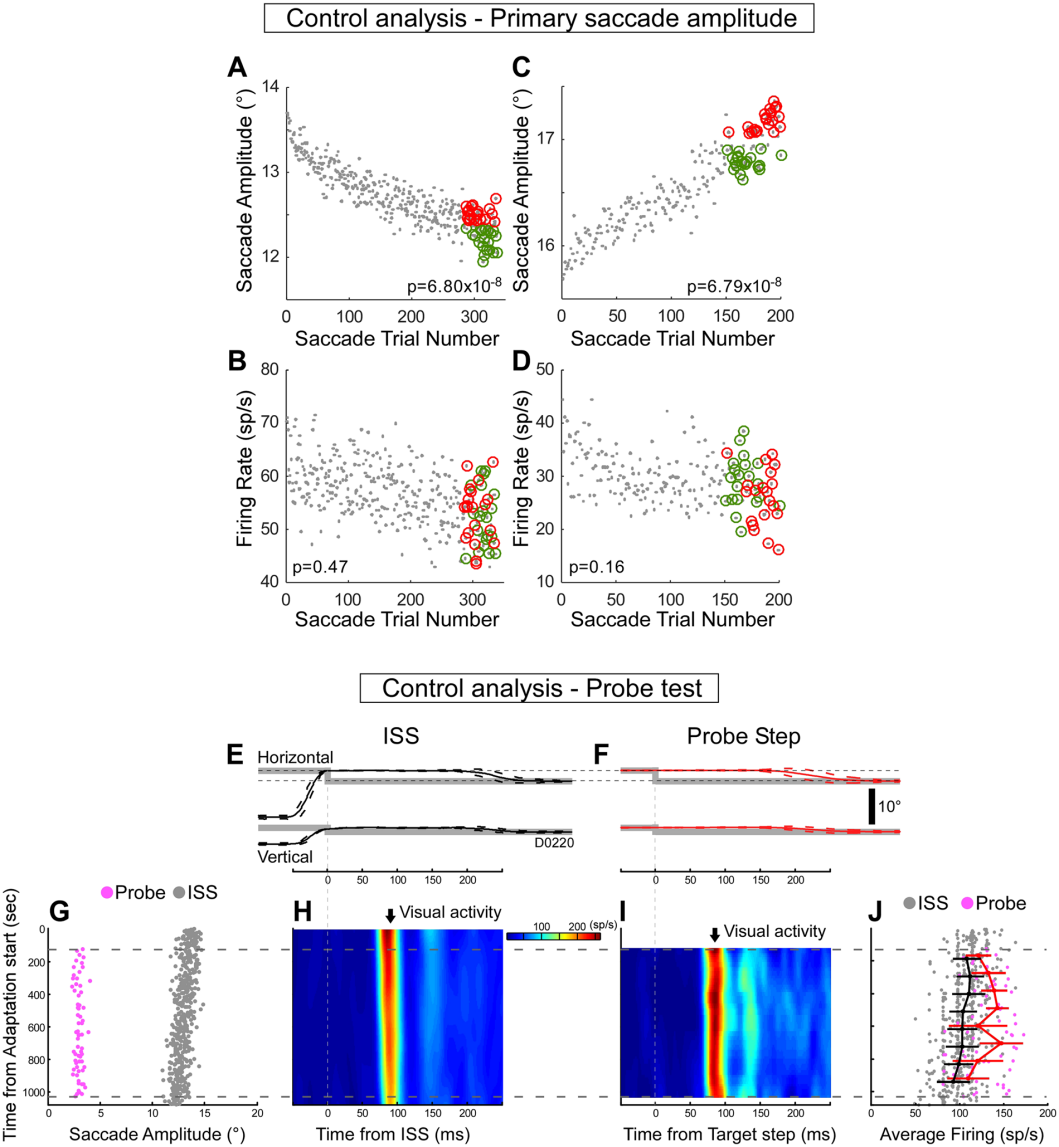
Control analyses. (**A**–**D**) Effect of primary saccade amplitude. (**A**,**B**) Population average during decrease adaptation. (**C,D**) Population average during increase adaptation. (**A**,**C**) 20 biggest (red) and smallest (green) saccades in the last 50 adaptation trials. (**B**,**D**) Visual activities for the selected saccades in A,C (red and green circles). (**E**–**J**) Test of visual probe inserted during amplitude decrease adaptation for a representative visual neuron. Comparison of eye movement responses when the same target step (grey) is used as an ISS (**E**) and a probe target (**F**); saccades aligned on the ISS or probe target step. In (**E**) eye movement (black) represents the average (±SD, broken lines) of the first 15 saccades during adaptation. In (**F**) eye movement (red) represents the average (±SD) of the first 15 probe step trials. Visual errors produced at ISS (**E**) and by the probe target step (**F**) are the same (horizontal dash lines). (**G**) The timing during adaptation of the ISS (grey dots) and the probe target steps (pink dots). Probe steps were inserted pseudo-randomly between ~100 to ~1000 sec after adaptation began. (**H**,**I**) Heat map of visual activity aligned on the ISS and single step, respectively. (**J**) Comparison of the changes in the visual responses for the ISS (grey dots, black line) and probe target steps (pink dots, red line). Black and red lines connect means (±SD) determined every 100 sec. Black lines are shifted slightly upward for ease of viewing.

Because not only the adaptation speed, but also the amplitude of the primary saccade changes during adaptation, we wondered whether the decrease in the visual activity during adaptation was related to the change in primary saccade amplitude. To test this possibility, we examined the visual activity associated with trials that had the largest (Fig. 7A, red circles) and smallest (green circles) 20 saccades among the last 50 trials in amplitude decrease adaptation of the population average data. Although the selected bigger and smaller amplitudes of the population average are significantly different (Wilcoxon rank sum test, p = 6.80 × 10^−8^, Fig. 7A), the average visual activities associated with the two amplitude groups were not (Wilcoxon rank sum test, p = 0.47, Fig. 7B). Like amplitude decrease adaptation, amplitude increase adaptation showed no significant difference in the average visual activity (Fig. 7D, Wilcoxon rank sum test, p = 0.16) for the two amplitude groups (Fig. 7C, Wilcoxon rank sum test, p = 6.79 × 10^−8^).

Furthermore, the visual activity decreased whether the amplitude decreased or increased, indicating that the change in the primary saccade is not related to the change in the visual activity. Therefore, we conclude that the amplitude of the primary saccade does not affect the visual activity.

### Control analysis – probe test

To address whether the decrease in visual activity is specific for the visual error that causes adaptation or occurs in response to all visual errors, we tested visual responses to simple probe target steps inserted during the course of adaptation. For another exemplar neuron illustrated in Fig. 7E–J, the visual errors at the end of the primary saccade and the following corrective saccades (Fig. 7E) and the single probe target steps and the responding saccades (Fig. 7F) are all of the same size. Figure 7G shows the timing of single probe targets (pink dots) during the amplitude decrease adaptation of primary saccades (grey dots); probe steps were inserted pseudo-randomly from ~100 to ~1000 sec after adaptation started. Although the visual activity for the ISS showed a clear qualitative decrease as adaptation progressed (Fig. 7H), the visual activity for the single probe step showed little qualitative change (Fig. 7I). A quantitative comparison of successive 100 sec bins throughout adaptation revealed that the visual response was significantly weaker to the ISS than to the probe step (Fig. 7J) (repeated measure of ANOVA, p (group) = 0.00022).

For the 15 amplitude decrease adaptations where we performed this quantitative comparison, the visual responses to the ISS during adaptation were significantly weaker than those to the probe step in 14 (93%) (repeated measure of ANOVA, p < 0.05). Therefore, we conclude that the decrease of the visual activity during adaptation is not caused by some sort of sensory habituation.

## Discussion

**Figure 8:**
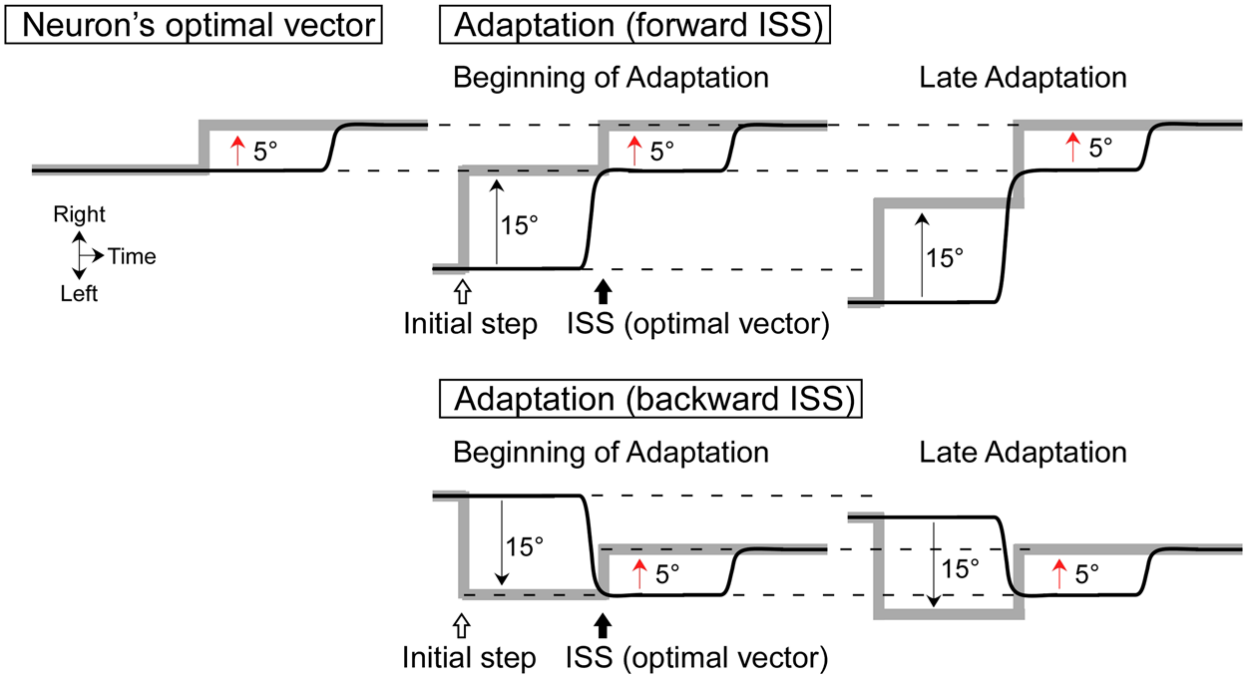
Schematic of target (gray lines) and eye (black lines) movements during adaptation for a unit with an optimal vector of 5° rightward. To induce adaptation, an intra-saccadic step (ISS) creates a rightward 5° visual error at the time of the primary saccade to an initial target step of 15° in both the rightward (forward ISS) and leftward (backward ISS) directions. To keep the visual error constant at 5° rightward during the entire adaptation session, the ISS size was continually increased (Late Adaptations).

Our objective was to determine whether the visual response of SC neurons decreased during adaptation as the error sensitivity decreased. Indeed, we found that the visual component of SC activity decreased significantly during adaptation (Figs 4 and 5). This decrease was due to neither a change in error size, which we held constant (see Methods, Fig. 8), nor the changing amplitude of the primary saccade (Fig. 7A–D). The decrease also was not the result of a general decrease of a neuron’s excitability over time, which, based on the constant saccade-related burst throughout adaptation for visuo-motor neurons, remained unchanged (Fig. 1). In addition, the decrease was not due to a sensory habituation because the visual response to probe target steps inserted during adaptation exhibited little change (Fig. 7E–J). Finally, it has been suggested that the decrease in SC visual activity might be the result of a shift of a neuron’s visual field on the SC map. We discuss this possibility in the supplemental material and suggest that this is highly unlikely (Supplementary Fig. S2). Therefore, we conclude that the sensitivity of SC neurons to the visual error decreases during adaptation.

A possible interpretation of our results is that the reduction of the visual sensitivity of SC neurons may decrease the sensitivity to the error that drives saccade adaptation. We realize that our data only show a correlation of SC visual activity with adaptation rate. However, we feel that the following studies that link the SC and the error signal that drives adaptation strongly suggest that this decreasing SC activity might actually provide a signal to help regulate the adaptation rate.

As we mentioned in the Introduction, appropriately timed electrical stimulation of the rostral SC during a saccade task produces saccade adaptation^19, 20^. The SC projects disynaptically to the oculomotor vermis (OMV) through the inferior olive, the source of complex spike activity in cerebellar P-cells^15–18^. Complex spike firing probability in the OMV encodes the direction and the size of the saccadic error^10–12^ associated with adaptation. Thus, the SC provides an input to the cerebellar pathway that is involved with saccade plasticity.

Several other published observations suggest that the error-related complex spike activity in the OMV could be influenced by SC activity. First, the timing of visual activity in the SC^21, 22^ and the enhancement of complex spike activity in the OMV during adaptation^10–12^ occur at about the same time, i.e., ~80–100 ms after a target and adapt step, respectively. Also, SC stimulation is most effective at producing adaptation when delivered within this time interval^19, 20^. Second, both SC visual activity^21, 24^ and complex spike activity^10–12^ occur only to contraversive target steps. The existence of these similar preferred directions is consistent with the known anatomical connections from the SC to the contralateral inferior olive^17, 18^ and then to the contralateral OMV^15, 16^. Third, the target amplitudes most effective at driving SC visual responses and complex spike activity in the OMV are small and show substantial overlap. The probability of complex spike occurrence is highest for ~3° steps in half of the population and <10° steps in the other half^11, 12^. Finally, stimulation of the rostral SC, which encodes smaller target amplitudes^19, 20^ is more effective at inducing adaptation than is stimulation of the caudal SC, which encodes larger target amplitudes^19, 20^. In this study, we obtained our data from the rostral SC and our neurons preferred smaller target amplitudes, ranging from 0.5 to 9° with a median of 4°. Thus, based on the totality of this evidence, we infer that the decrease in the SC visual activity during adaptation very likely influences the decrease in the rate of adaptation.

Although the probability of complex spike activity is related to the error signal driving adaptation, there is no evidence yet of a complex spike signal related to the rate of adaptation. In this study, we used a fixed error paradigm to reveal an SC visual sensitivity to the error that occurs during such adaptation. Thus far, a similar fixed adaptation error paradigm has not been used to study complex spike activity. Instead, the sensitivity to error in the OMV has been documented only with a variable adaptation error signal that decreased with adaptation^2, 10^. However, it seems likely that complex spike activity might carry a rate of adaptation signal because the sensitivity to error is low in patients with cerebellar degeneration^25^.

If a weaker SC signal produces slower adaptation, how might it do so? One attractive possibility is that the weaker SC activity causes the complex spikes driven by the adaptation error to occur with a lower probability. Alternatively, the complex spikes could be shortened (fewer spikelets), which could lead to less calcium entering P-cells. This weaker entry in turn could produce a slower plastic change and a slower adaptation. A shorter complex spike was suggested to account for the role of the flocculus in smooth pursuit learning^26^.

It should be pointed out that a reduction of SC visual sensitivity is not the only process that could lead to the slowing of saccade adaptation. Even when adaptation is induced by a constant stimulation of the rostral SC so there presumably is no change in the SC output, adaptation still gradually slows to a soft asymptote^19, 20^. This result suggests that there is a mechanism to slow saccade adaptation that isn’t driven by the SC. One speculation suggested in studies of long-term saccade adaptation is that the capacity for change in short-term adaptation, which most likely occurs through plasticity in the OMV, is limited, causing adaptation eventually to slow down or stop^27^.

Thus far, we have considered how SC visual activity could influence downstream structures and saccades. What factors unrelated to error could modulate SC visual sensitivity during adaptation? Several possibilities have been suggested by previous studies; we will consider the 5 most likely here. [1] The visual activity of visuo-motor neurons is greater for short latency, i.e. express saccades, than for normal latency saccades^22, 23, 28, 29^. This cannot be a factor here because the latency of corrective saccades was always >150 ms, which is within the range of the normal latency saccades. [2] Saccades that occur near a target step suppress the visual activity for the target step^30^. Again, this is not a concern because the primary saccade always occurred at the ISS during adaptation. [3] Target uncertainty influences SC visual activity^31, 32^, but there was no target uncertainty during our adaptations. Finally, there are two factors that involve attention and motivation. [4] SC visual activity is higher for a target step that requires a subsequent saccadic response than one that doesn’t, presumably because attention is focused there^33–35^. Also, SC visual activity is less if the target appears at a previously notified location than at an un-notified location (“inhibition of return”^29, 36–38^). [5] SC visual activity is modulated by reward-related motivation^39^. Although we did not control either the monkey’s attention or motivation intentionally by giving notification of or changing the reward conditions, attention or motivation might have been affected by a mental fatigue during adaptation because the task was repetitious^40^. We have no way of assessing whether the monkey’s mental state changed over time, but such internal changes during adaptation might have reduced the SC visual activity and, in turn, the complex spike activity, leading to slower adaptation.

Because the visual error signal for adaptation was constant in our experiments, the slowing of adaptation reflects a reduction in the sensitivity to the error signal in driving adaptation. Other studies have defined the sensitivity to error as the amount by which a response changes in the trial following an error^5, 6^. In the monkey, the sensitivity to an error in smooth pursuit apparently was demonstrated from one trial to the next where each eye movement shows a small, learned deflection in the direction of the error from the prior trial^26^. When we performed a similar trial by trail analysis on our data, the variability of each response in our paradigm made it impossible to detect an error sensitivity.

In our calculations, we used an exponential to fit the adaptation curve, because it is the consensus fit in the literature^3, 41, 42^. It shows that adaptation occurred faster at the beginning and slower at the end. We also have tried to document the adaptation rate throughout the entire adaptation by taking the derivative of some smoothing non-linear fit, using numerical differentiation of non-smoothed data^43^, or taking the slope of a linear regression fit to the data. However, to use these approaches, it is necessary to make some arbitrary assumptions, e.g., the smoothing parameters of the procedure or how many trials to fit in a linear regression. Therefore, we confined our analysis to the beginning and end of adaptation where both the firing rates and adaptation speeds were clearly different.

## Methods

Four male *MacacaMulatta* monkeys served as subjects. A previous study^9^ describes our surgical and recording procedures in detail. Briefly, we implanted each monkey with fixtures to prevent head movements, an eye coil to measure eye position^40, 41^ and a recording chamber aimed at the midline between the left and right superior colliculus (SC) and attached to the skull.

Animals were rewarded with applesauce to track a 0.3° projected laser spot in a dimly-lit, sound-attenuating booth. Rewards were delivered when their gaze remained within ±2° windows of the horizontal and vertical components of target position continually for at least 0.5 sec. The targeting saccade had to occur within 0.6 sec of the target step and the subsequent fixation maintained for at least 0.3 sec.

After a monkey reliably tracked the target, we made penetrations with a glass coated tungsten micro-electrode (Alpha-Omega, Alpharetta, USA) through a guide tube.

All experiments were performed in accordance with the Guide for the Care and Use of Laboratory Animals and exceeded the minimal requirements recommended by the Institute of Laboratory Animal Resources and the Association for Assessment and Accreditation of Laboratory Animal Care International. All the procedures were evaluated and approved by the local Animal Care and Use Committee of the University of Washington.

In each experiment, we isolated a SC neuron while the monkey made saccades to target steps with pseudorandom vectors. To identify the neuron’s optimal vector, we first examined the burst associated with target steps and saccades angled every 10° and determined the angle halfway between target step directions where the burst was the weakest^21, 24^. In the optimal direction, we then examined the burst for amplitudes that varied in 0.5° increments and determined the optimal amplitude with the strongest burst^44, 45^. Because stimulation of the rostral SC induces greater adaptation than stimulation of the caudal SC^19, 20^, we focused on the rostral SC, whose neurons respond best to small target and/or saccade amplitudes. The optimal amplitudes in our SC sample ranged from 0.5–9° (median = 4.0°). To identify whether SC neurons were visual, visuo-motor, saccade-related burst, or buildup^46, 47^, we used the overlap paradigm for the neuron’s optimal vector. In this paradigm, the monkey saccades to a new spot after the fixation spot is extinguished (0.8 sec overlap)^35, 48^.

Next we recorded unit activity as the monkey underwent saccade adaptation. Pre-adaptation data were collected for saccades to 15° target steps along the neuron’s optimal axis. The target stepped randomly along the optimal axis within ±18° of straight-ahead. We collected ~100 saccades for target steps in the optimal and opposite directions. Then, we adapted saccade amplitude in both directions simultaneously. For example, the optimal vector for the neuron in Fig. 8 was 5° rightward. To produce a gain increase adaptation, primary saccades to a 15° rightward target were followed by a rightward 5° intra-saccadic step (forward-ISS). Primary saccades to 15° leftward targets also were followed by a rightward 5° (backward) ISS, causing a gain decrease adaptation. To keep the post-saccadic visual error constant at the neuron’s optimal 5° vector during the adaptation, we modified the traditional paradigm^2^. We detected the eye position at the end of the saccade (when velocity dropped below 20°/s) and moved the target to create a 5° visual error from that location^3, 4^ (Fig. 8, Late Adaptations). Consequently, the amplitude of corrective saccades also remained constant at ~5°. To begin the next trial, the target stepped 15° rightward or leftward from that position; therefore, the next start position was random for these trials and the interleaved probe trials (see below).

We recorded the activity of 49 neurons during amplitude increase and decrease adaptation. For 15, we also tested SC activity for saccades to probe target steps equal to the neuron’s optimal vector during adaptation. We inserted the probe trials randomly during adaptation (10% of all trials), so the start position was random. These data controlled whether the change in visual response during adaptation occurred only when the visual target step served as an ISS during adaptation or also when it only elicited a saccade.

We digitized eye and target position signals at 1 kHz and sampled unit activity at 50 kHz using Power1401 data acquisition/controller hardware (CambridgeElectronicDesign, Cambridge, UK). We saved data to a hard disk for later analysis. A custom program running in Spike2 (CambridgeElectronicDesign, Cambridge, UK) controlled target movement and the monkey’s reward via the Power1401 hardware.

A custom program running in Spike2 analyzed the saved data. It detected the occurrence of a saccade when eye velocity exceeded 75°/s within 70–800 ms after a target jump and marked saccade onset and end when vector eye vector exceeded or fell below 20°/s, respectively. The program measured saccade amplitude, peak velocity and duration, and the target distance before each saccade. A voltage threshold detected each action potential and the program saved its time of occurrence. The marked events were carefully checked by eye and if a unit’s isolation became problematic, we discarded it. All 49 neurons used in this study were well isolated during the entire pre- and adaptation sessions. The saccade attributes, target positions, and action potential times were exported to MATLAB (MathWorks, Natick, USA). Saccades whose initial eye positions differed from initial target positions by >5° were not analyzed. To analyze the SC activity, we created a spike density function (SDF) by replacing each action potential with a Gaussian function (4 ms SD) centered on the time of spike occurrence^21^.

To classify the neuron types as visual, visuo-motor, saccade-related burst and buildup, we divided the activity obtained with the overlap paradigm into four intervals and calculated the mean discharge rates in each^35^. From the target step (0 ms), the base line interval ranged from −200 ms (before) to 0, the visual interval from 0 to +200 ms and the delay interval from the +300 to 800 ms. The saccade interval encompassed the 50 ms before saccade onset. If the mean rate was significantly greater during the visual than the baseline intervals (paired t-test, p < 0.05) but not during the saccade interval, the cell was considered a visual neuron. If the mean rate was significantly greater than the baseline only during the saccade interval, it was a saccade-related burst neuron. A neuron that showed significantly greater discharges for both the visual and saccade intervals, but not the delay interval, was a visuo-motor neuron. Finally, neurons with a significantly greater discharge during the delay interval were categorized as buildup neurons. For our 49 SC neurons, 14 were visuo-motor, 19 were visual, 12 were saccade-related burst and 4 were buildup. This distribution of neuron types does not represent the actual SC population because we always tested the first neuron isolated.

In our SC penetrations, we usually encountered pure visual neurons most dorsally, neurons with both visual and saccade sensitivities more ventrally and finally neurons that discharged bursts related only to saccades most ventrally. This dorso-ventral encounter sequence has been reported by others who located the pure visual neurons in the superficial layer of the SC and those with motor related activity in the deeper layers^46^.

For each saccade trial during adaptation, we measured visual activity for visual, visuo-motor and buildup neurons and saccade-related activity for visuo-motor, saccade-related burst and buildup neurons. To document the visual activity, we determined the average magnitude of the SDF between 50 and 100 ms^21, 22^ after the onset of an ISS across all trials. To document the saccade-related burst activity, we determined the average magnitude of the SDF between 8 ms before until 8 ms^21^ after each corrective saccade.

To visualize the neuronal discharge, we smoothed the SDFs across trial number along the vertical axis by a moving average with a span the size of a bin width (25 trials) and presented the data as a surface plot.

To analyze the changes in saccade amplitude and unit activity during adaptation, we plotted them against saccade trial number. To display the course of adaptation, we fitted the saccade amplitude with a two-term exponential function.

$${\rm{Saccade}}\,{\rm{Amplitude}}={\rm{a}}\times \exp ({\rm{b}}\times {\rm{Trial}}\,{\rm{Number}})+{\rm{c}}\times \exp ({\rm{d}}\times {\rm{Trial}}\,{\rm{Number}})$$

To measure the course of the firing rate during adaptation, we calculated the average firing rate for each trial. We then compared the average firing rate for the first and last 50 saccades (Wilcoxon rank sum test). The change in firing rate during adaptation was summarized for each neuron individually and as a population average.

To test whether changes in primary saccade amplitude during adaptation accounted for the changes in visual activity, we tested the sensitivity of visual activity to primary saccade amplitude at the end of the adaptation. We selected the largest and smallest 20 saccades from the last 50 saccades during adaptation and compared their visual activities (Wilcoxon rank sum test).

We tested visual responses to target steps of the optimal vector that served either to produce adaptation or did not, i.e. a probe trial not linked with a previous primary saccade. We plotted the visual activities against the saccade’s time during adaptation for both the ISS and the probe trials and tested whether they were significantly different (repeated-measure of ANOVA).

The datasets of this study are available from the corresponding author upon request.

## Electronic supplementary material

Supplementary 1 and 2
