# Supplementary material for: Change in sensitivity to visual error in superior colliculus during saccade adaptation
Source: Sci Rep. 2017 Aug 29;7:9566. doi: 10.1038/s41598-017-10242-z (PMC5574973; doi:10.1038/s41598-017-10242-z)
Supplement: Supplementary file 1 — Supplementary 1 and 2 [file 41598_2017_10242_MOESM1_ESM.pdf]

Change in sensitivity to visual error in superior colliculus during saccade adaptation.

Yoshiko Kojima and Robijanto Soetedjo

Supplementary Fig. S1

Control analysis – visual error

Although, we controlled the size of the ISS to make the visual error constant during adaptation, the error nevertheless changed by a very small amount. However, this small change in visual error does not explain the change in the visual activity during adaptation (i.e., Fig. 1D). Consider the data in Fig. S1A-D, which are extracted from the experiment illustrated in Fig. 1. During adaptation, the visual error decreased slightly by 0.06° for this unit (Fig. S1A); the median amplitude of the visual error for the first (4.10° and 0.05°, median and interquartile range, respectively; Fig. S1B, blue broken line) and last 50 saccades (4.04° and 0.07°, median and interquartile range, respectively; Fig. S1B, orange broken line) was significantly different (Wilcoxon rank sum test, p =1.7×10-5). Fig. S1C shows a plot of the visual field of this neuron. A polynomial fit estimated that the average firing rate for a 4.10° visual error was 196.4 spikes/s (Fig. S1C, D, blue broken line) and 195.4 spikes/s for a 4.04° visual error (Fig. S1C, D, orange broken line), an average difference of only 1 spike/s. The change in firing rate for this very small change of the visual error cannot account for the ~28 spikes/s change of visual activity that occurs during adaptation (Fig. 1D).

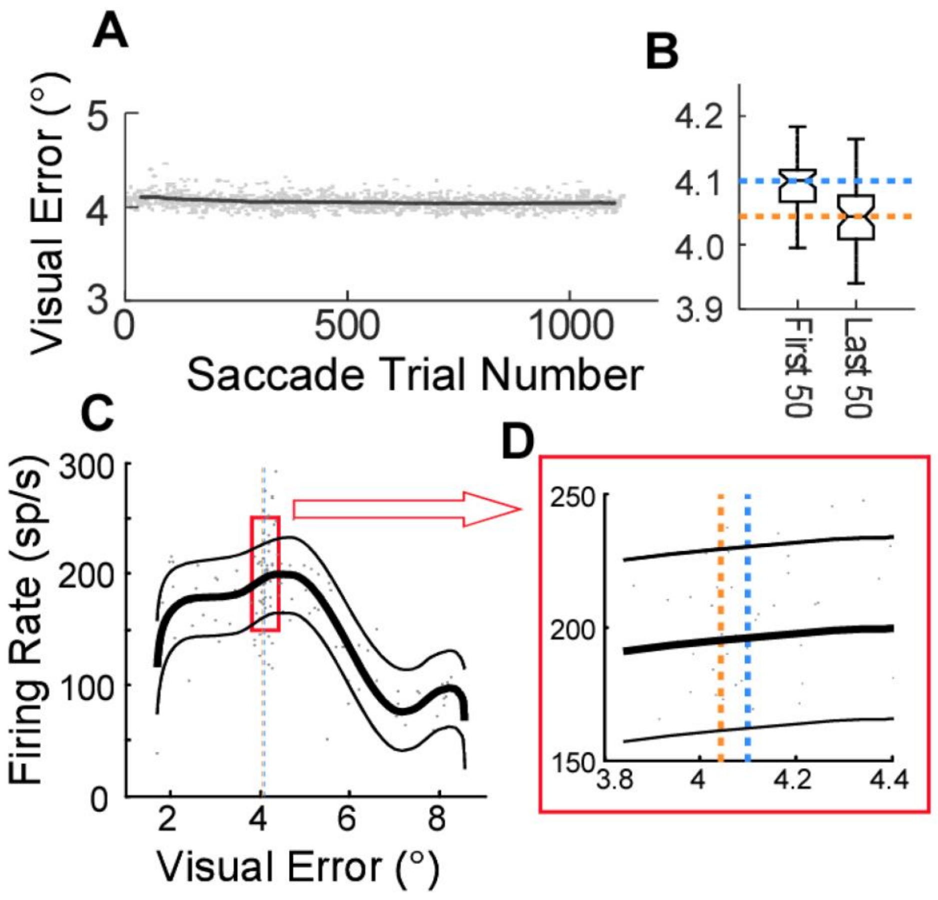

Fig. S1

Control analysis for changes in visual error for a representative neuron. **A**, visual error size during the entire adaptation session. Black line is a moving average of 250 saccades. **B**, average visual error for first and last 50 saccades. Blue and orange broken lines indicate the median of the first and last 50 saccades, respectively. **C**, visual response field of this neuron. Thick black line indicates a 6th polynomial fit. Black thin lines indicate the standard deviation of the fit. **D**, detail of the red box in C. Blue and orange broken lines indicate the median of the first and last 50 saccades, respectively.

Supplementary Fig. S2

We found that many neurons in the superior colliculus (SC) exhibited a decrease in visual activity during saccade adaptation. This decrease could be the result of two different mechanisms. One possibility is that the visual response field of a neuron collapses so the peak response (Fig. S2A, red arrow) is less for the same visual error (blue arrow) after adaptation. This would be considered a decrease in visual sensitivity. The other is that the visual response field retains its shape but is shifted to another vector representation on the SC map (Fig. S2B, red arrow). In this case, the neuronal activity at the measured error amplitude (green line) would also decrease (blue arrow).

Fig. S2C (neuron in Fig. 1) and D (neuron in Fig. 2) show the visual fields in the direction of the unit's preferred vector for two representative neurons that showed a decrease in visual activity during adaptation. For the neuron in Fig. S2C, a polynomial fit estimated that the field's peak amplitude before adaptation was 4.5°. The average visual error of the ISS that drove adaptation was 3.98° ± 0.13 (green vertical line with green shade), which is smaller than the peak amplitude of the visual field. For the neuron in Fig. S2D, the average visual error that drove adaptation was 2.95° ± 0.22, which is larger than the field's peak amplitude (2.5°). If the visual field shifts *rightward* (red arrows), the activity at the measured amplitude (vertical green line with green shade) in C should decrease and D should increase (blue arrows). However, the activities of both neurons in fact decreased during adaptation. If the fields were to shift to shift *leftward* (no arrows shown), the neuron in C would experience an increase in activity and the unit in D a decrease in activity. Thus, this analysis rejects the possibility that the reduction of the visual activity during adaptation is always caused by the same shift of the visual field.

It is possible that adaptation would be accompanied by a rightward receptive field shift for some neurons and a leftward for others, whichever direction would produce a decrease in visual activity as we found for 74% of our neurons. Although it seems highly unlikely, we have no data to reject this possibility.

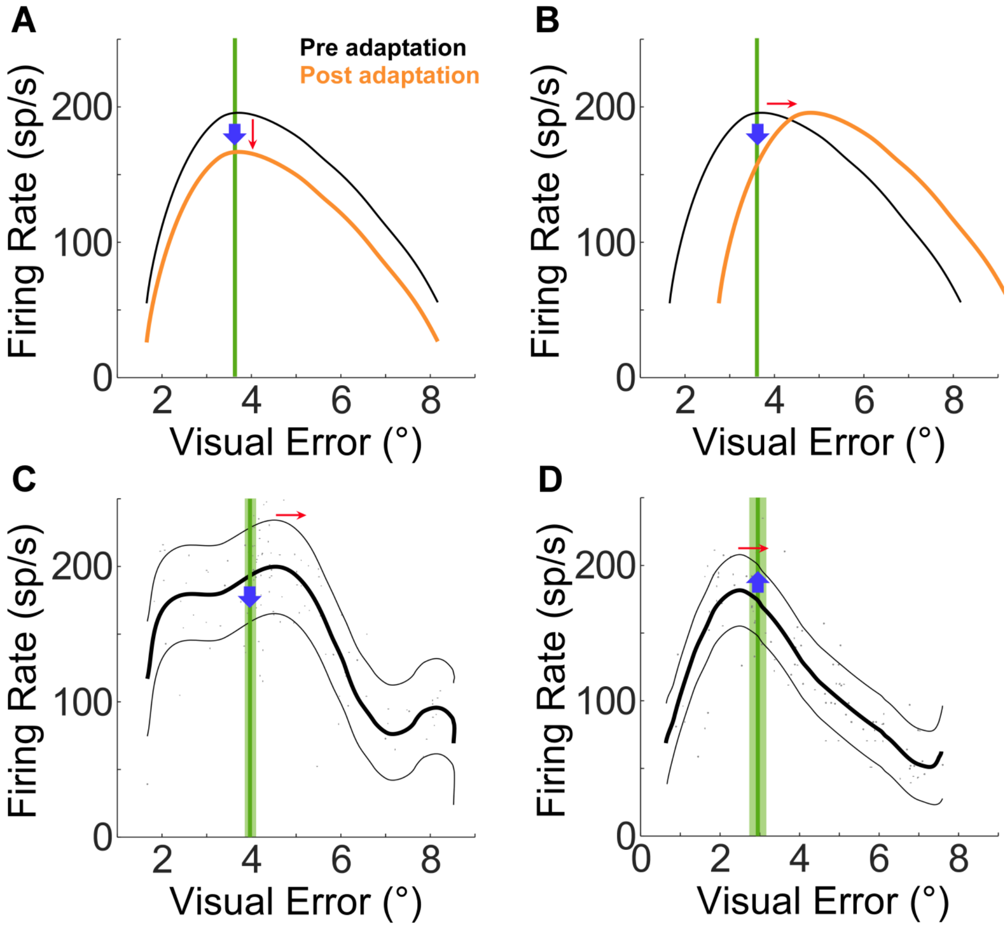

Fig. S2
Two mechanisms that could reduce SC visual activity during adaptation. **A, B**, Schematic visual response fields demonstrate a sensitivity decrease (A) and a visual field shift (B). Black and orange lines indicate the visual field before and after adaptation, respectively. Red arrow indicates the direction of the field shift. Blue arrow (downward is decrease) indicates the expected change in neuronal activity due to a peak field decrease (A) and to a field shift (B). **C, D**, Visual response fields of the neurons in Fig. 1 and Fig. 2, respectively. Thick black line indicates a 6 polynomial fit (±1SD; thin lines) to the activity measured during adaptation. Green line with green shading indicates the average visual error (± 1SD) exposed during adaptation, i.e., by the ISI.
